# Supplementary figures and images for: Identification and predictive machine learning models construction of gut microbiota associated with lymph node metastasis in colorectal cancer
Source: mSystems. 2025 Jul 8;10(8):e00339-25. doi: 10.1128/msystems.00339-25 (PMC12363233; doi:10.1128/msystems.00339-25)

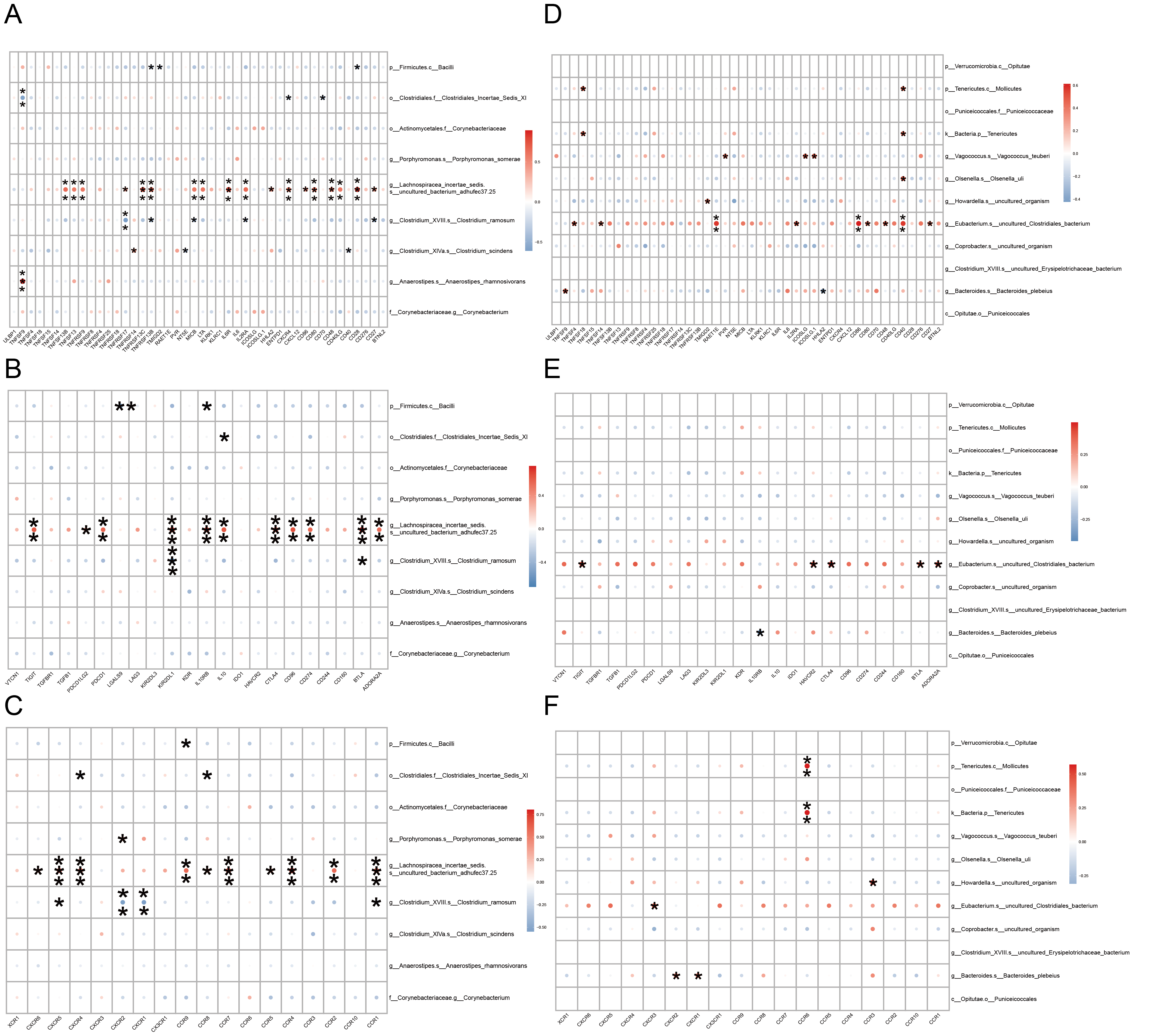

Supplement: Fig. S1 — Heat map of correlation between dominant bacteria and immune activation genes, immune suppressor genes, and chemokine receptors in NLNM and LNM groups. [file msystems.00339-25-s0001.tif]

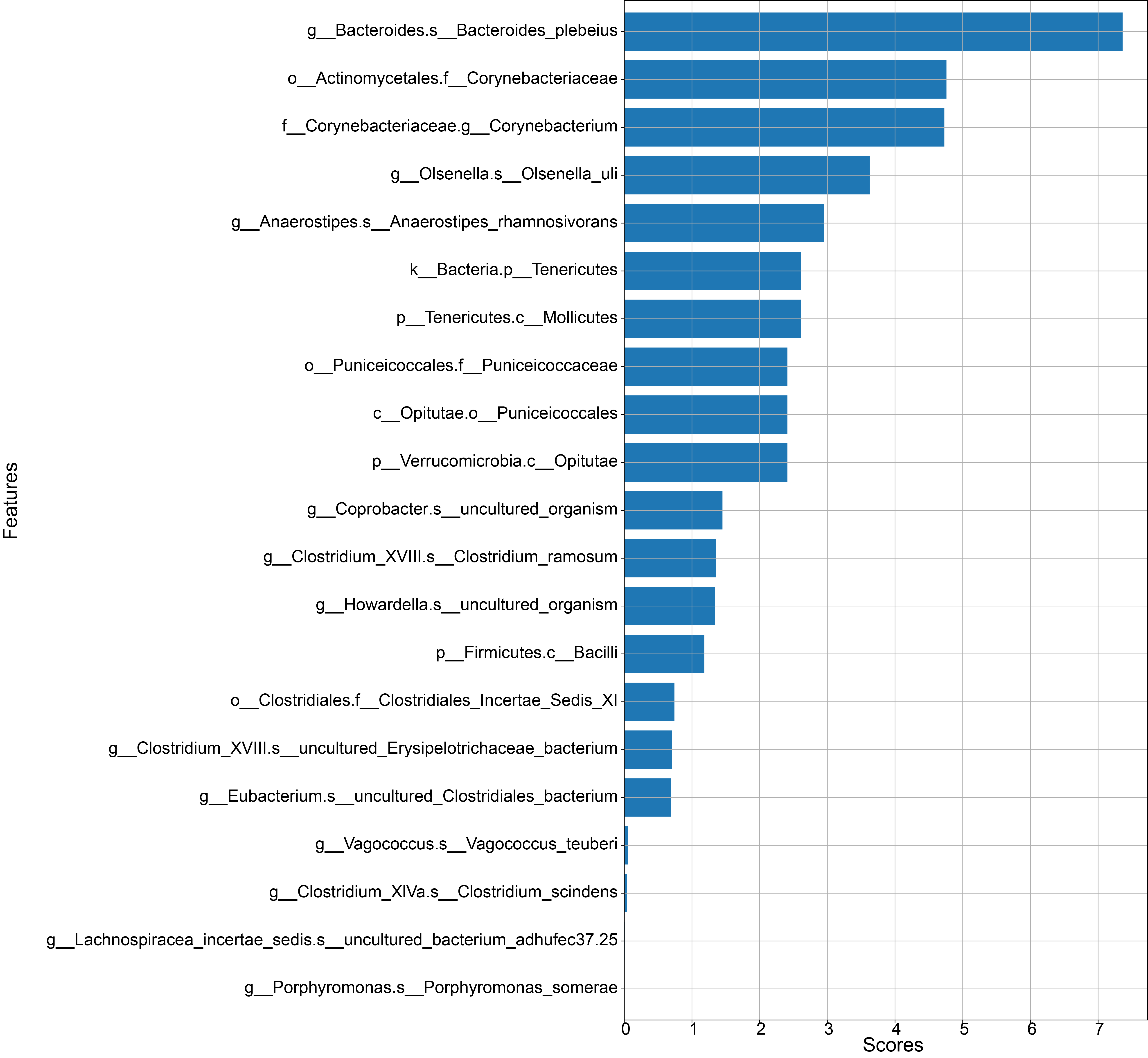

Supplement: Fig. S2 — Ranking the importance of gut microbiota associated with LNM to predict lymph node status in CRC patients. [file msystems.00339-25-s0002.tif]
