# Supplementary material for: Identification and predictive machine learning models construction of gut microbiota associated with lymph node metastasis in colorectal cancer
Source: mSystems. 2025 Jul 8;10(8):e00339-25. doi: 10.1128/msystems.00339-25 (PMC12363233; doi:10.1128/msystems.00339-25)
Supplement: Legends — for supplemental figures and tables. [file msystems.00339-25-s0003.docx]

**Supplemental Figures**

**Fig.S1A. Heat map of correlation between dominant bacteria and immune activation genes in NLNM.**

**Fig.S1B. Heat map of correlation between dominant bacteria and immune suppressor genes in NLNM.**

**Fig.S1C. Heat map of correlation between dominant bacteria and chemokine receptors in NLNM.**

**Fig.S1D. Heat map of correlation between dominant bacteria and immune activation genes in LNM.**

**Fig.S1E. Heat map of correlation between dominant bacteria and immune suppressor genes in LNM.**

**Fig.S1F. Heat map of correlation between dominant bacteria and chemokine receptors in LNM.**

Horizontal coordinate is gene, vertical coordinate is colony, red represents positive correlation, blue represents negative correlation, color depth represents Pearson correlation coefficient size, color from light to dark indicates Pearson correlation coefficient value from small to large**.** The "*" in the graph represents the size of the p-value: No * for P-value ≥ 0.05, * for 0.01 ≤ P<0.05, ** for 0.001 ≤ P<0.01, *** for P<0.001.

**Fig.S2. Ranking the importance of gut microbiota associated with LNM to predict lymph node status in CRC patients.** The vertical axis represents the gut microbiota associated with LNM that are features in the Random Forest (RF) model and the Multilayer Perceptron (MLP) model. The horizontal axis of the graph displays the score that quantifies the importance of the feature; the higher the score, the greater the importance of the feature.

**Supplemental Tables**

**Table.S1. Results of LEfSe analysis.**

Taxonomy: LNM-related gut microbiota information; Group: group with significant abundance of differential species; LDA: effect value of LNM-associated gut microbiota after log10 treatment; species with LDA scores (log10) greater than 2 and p-values less than 0.05 are shown in Table.

**Table.S2.** **KEGG pathways in the gut microbiota of CRC patients in LNM and NLNM.**

KEGG_id: description: KEGG pathway; Mean in NLNM: the predicted abundance value of this pathway in each sample in NLNM; Mean in LNM: the predicted abundance value of this pathway in each sample in LNM.

**Table.S3. List of differential GO items and KEGG pathways of LNM and NLNM.**

GO items: Enriched GO entries. KEGG pathways: Enriched KEGG entries. LogFC: FC represents the folding change, that is, the ratio of the expression of LNM and NLNM. The logarithm is taken as the base of 2. Statistically significant when p-value is less than 0.05.

**Table.S4. Association between LNM-associated GO and KEGG enrichment and the dominant gut microbiota in LNM and NLNM.**

The r.value is the Spearman correlation coefficient value. P-value less than 0.05 is statistically significant.
