# Supplementary material for: Identification and predictive machine learning models construction of gut microbiota associated with lymph node metastasis in colorectal cancer
Source: mSystems. 2025 Jul 8;10(8):e00339-25. doi: 10.1128/msystems.00339-25 (PMC12363233; doi:10.1128/msystems.00339-25)
Supplement: Table S1 — Results of LEfSe analysis. [file msystems.00339-25-s0004.docx]

**Table.S1. Results of LEfSe analysis**

| **Taxonomy** | **Group** | **LDA** | **Pvalue** |
| --- | --- | --- | --- |
| g__Clostridium_XlVa.s__Clostridium_scindens | NLNM | 3.507 | 0.0008 |
| o__Actinomycetales.f__Corynebacteriaceae | NLNM | 3.086 | 0.0025 |
| f__Corynebacteriaceae.g__Corynebacterium | NLNM | 3.087 | 0.0027 |
| g__Clostridium_XVIII.s__Clostridium_ramosum | NLNM | 4.083 | 0.0242 |
| p__Firmicutes.c__Bacilli | NLNM | 5.33 | 0.0294 |
| g__Anaerostipes.s__Anaerostipes_rhamnosivorans | NLNM | 2.35 | 0.0317 |
| o__Clostridiales.f__Clostridiales_Incertae_Sedis_XI | NLNM | 4.762 | 0.0353 |
| g__Porphyromonas.s__Porphyromonas_somerae | NLNM | 3.697 | 0.0437 |
| g__Lachnospiracea_incertae_sedis.s__uncultured_bacterium_adhufec37.25 | NLNM | 3.057 | 0.0495 |
| g__Bacteroides.s__Bacteroides_plebeius | LNM | 4.018 | 0.0075 |
| g__Eubacterium.s__uncultured_Clostridiales_bacterium | LNM | 3.762 | 0.0152 |
| g__Howardella.s__uncultured_organism | LNM | 2.528 | 0.0179 |
| g__Coprobacter.s__uncultured_organism | LNM | 3.573 | 0.0191 |
| g__Olsenella.s__Olsenella_uli | LNM | 2.647 | 0.0224 |
| p__Tenericutes.c__Mollicutes | LNM | 2.801 | 0.033 |
| p__Verrucomicrobia.c__Opitutae | LNM | 3.362 | 0.033 |
| c__Opitutae.o__Puniceicoccales | LNM | 3.432 | 0.033 |
| o__Puniceicoccales.f__Puniceicoccaceae | LNM | 3.196 | 0.033 |
| k__Bacteria.p__Tenericutes | LNM | 2.822 | 0.033 |
| g__Vagococcus.s__Vagococcus_teuberi | LNM | 3.058 | 0.0387 |
| g__Clostridium_XVIII.s__uncultured_Erysipelotrichaceae_bacterium | LNM | 3.417 | 0.0494 |

Footnote: Taxonomy: information of LNM-associated gut microbiota; Group: CRC patients with LNM and NLNM; LDA (log10): effect value of LNM-associated gut microbiota, which was after log10 transformation; the species showed in the table were selected by the threshold of |LDA score| >2 and P < 0.05. P value: P<0.05 as statistical significance.
