# Supplementary material for: Identification and predictive machine learning models construction of gut microbiota associated with lymph node metastasis in colorectal cancer
Source: mSystems. 2025 Jul 8;10(8):e00339-25. doi: 10.1128/msystems.00339-25 (PMC12363233; doi:10.1128/msystems.00339-25)
Supplement: Table S2 — KEGG pathways in the gut microbiota of CRC patients in LNM and NLNM. [file msystems.00339-25-s0005.docx]

**Table.S2. KEGG pathways in the gut microbiota of CRC patients in LNM and NLNM**

| KEGG_id: description | Mean In NLNM | Mean In LNM | Pvalue |
| --- | --- | --- | --- |
| ko04614:Renin-angiotensin system | 7971.462158 | 0 | 0.01621 |
| ko00100:Steroid biosynthesis | 39976566.13 | 12422728.36 | 0.02803 |
| ko04144:Endocytosis | 663132.666 | 536832.5452 | 0.03293 |
| ko05110:Vibrio cholerae infection | 54948.87514 | 42401.71751 | 0.03421 |
| ko03040:Spliceosome | 59721.98935 | 16288.73143 | 0.05313 |
| ko05410:Hypertrophic cardiomyopathy (HCM) | 1079810.294 | 814178.6583 | 0.06715 |
| ko03450:Non-homologous end-joining | 22991286.55 | 19425302.48 | 0.1085 |
| ko00361:Chlorocyclohexane  and chlorobenzene degradation | 97600308.62 | 86870621.99 | 0.11022 |
| ko00511:Other glycan degradation | 3245547211 | 2110170440 | 0.11551 |
| ko00040:Pentose and glucuronate interconversions | 1079373817 | 778441233.8 | 0.12572 |
| ko00791:Atrazine degradation | 99532675.04 | 98020781.54 | 0.12716 |
| ko00053:Ascorbate and aldarate metabolism | 389275538.7 | 317679100.4 | 0.12765 |
| ko00562:Inositol phosphate metabolism | 308286184.3 | 239842561.1 | 0.12862 |
| ko04621:NOD-like receptor signaling pathway | 146150941.3 | 89446094.32 | 0.1296 |
| ko00590:Arachidonic acid metabolism | 289770.5017 | 0 | 0.13385 |
| ko00121:Secondary bile acid biosynthesis | 1783472957 | 1215669811 | 0.13458 |
| ko00500:Starch and sucrose metabolism | 1735088244 | 1301649337 | 0.13764 |
| ko00120:Primary bile acid biosynthesis | 198441266.5 | 135944328.4 | 0.13867 |
| ko00480:Glutathione metabolism | 684705848 | 520037016.5 | 0.14286 |
| ko05120:Epithelial cell signaling in  Helicobacter pylori infection | 254073924.6 | 179936211.5 | 0.14392 |
| ko00051:Fructose and mannose metabolism | 1682539947 | 1205015356 | 0.14499 |
| ko00780:Biotin metabolism | 2554150572 | 1616646043 | 0.14499 |
| ko00521:Streptomycin biosynthesis | 2886684021 | 1946614357 | 0.14714 |
| ko00600:Sphingolipid metabolism | 900031755.9 | 605868625 | 0.14932 |
| ko00030:Pentose phosphate pathway | 2508003496 | 1845177528 | 0.15263 |
| ko00520:Amino sugar and nucleotide sugar metabolism | 1812283544 | 1251506233 | 0.15263 |
| ko00910:Nitrogen metabolism | 957355555.9 | 700401397 | 0.156 |
| ko00633:Nitrotoluene degradation | 463127285.4 | 380871471.5 | 0.15714 |
| ko01055:Biosynthesis of vancomycin group antibiotics | 3931414550 | 2516056569 | 0.15714 |
| ko02010:ABC transporters | 966888423.9 | 770409233.5 | 0.15714 |
| ko00730:Thiamine metabolism | 2533372378 | 1743147612 | 0.15828 |
| ko04122:Sulfur relay system | 1520135541 | 1100376050 | 0.15828 |
| ko00860:Porphyrin and chlorophyll metabolism | 1048194018 | 717650582.4 | 0.15943 |
| ko02060:Phosphotransferase system (PTS) | 551364260 | 520484536.3 | 0.15943 |
| ko00052:Galactose metabolism | 1848441902 | 1381810596 | 0.16058 |
| ko00561:Glycerolipid metabolism | 711108982 | 568039134.1 | 0.16058 |
| ko00627:Aminobenzoate degradation | 213665813.5 | 149140905.6 | 0.16058 |
| ko00450:Selenocompound metabolism | 1710346780 | 1158121328 | 0.16174 |
| ko00280:Valine, leucine and isoleucine degradation | 631804970.9 | 409550096.6 | 0.16291 |
| ko00310:Lysine degradation | 278992857.4 | 184868999.6 | 0.16291 |
| ko00350:Tyrosine metabolism | 393472912.1 | 296547673.8 | 0.16291 |
| ko00510:N-Glycan biosynthesis | 83772029.07 | 56564836.3 | 0.16291 |
| ko00908:Zeatin biosynthesis | 1124384800 | 702845863.1 | 0.16291 |
| ko00010:Glycolysis / Gluconeogenesis | 1746784292 | 1254313213 | 0.16408 |
| ko00650:Butanoate metabolism | 1034009994 | 740113731.9 | 0.16526 |
| ko00250:Alanine, aspartate and glutamate metabolism | 2628973428 | 1848615689 | 0.16644 |
| ko00473:D-Alanine metabolism | 2673542894 | 1799510148 | 0.16644 |
| ko00710:Carbon fixation in photosynthetic organisms | 2460485203 | 1722329187 | 0.16644 |
| ko00960:Tropane, piperidine and  pyridine alkaloid biosynthesis | 595809226.3 | 431450937.3 | 0.16644 |
| ko04962:Vasopressin-regulated water reabsorption | 15789.41825 | 17885.97391 | 0.16765 |
| ko00270:Cysteine and methionine metabolism | 1904408249 | 1375947942 | 0.16884 |
| ko00471:D-Glutamine and D-glutamate metabolism | 3193091605 | 2220212670 | 0.16884 |
| ko00760:Nicotinate and nicotinamide metabolism | 1728225154 | 1198144549 | 0.16884 |
| ko00430:Taurine and hypotaurine metabolism | 1045664285 | 734663190.3 | 0.17004 |
| ko00790:Folate biosynthesis | 2041777395 | 1324491089 | 0.17004 |
| ko00360:Phenylalanine metabolism | 433112062.9 | 342147459.1 | 0.17125 |
| ko00440:Phosphonate and phosphinate metabolism | 212060337.2 | 159486450.8 | 0.17125 |
| ko03013:RNA transport | 80883622.74 | 59838506.57 | 0.17125 |
| ko03430:Mismatch repair | 2679758916 | 1850403972 | 0.17125 |
| ko03440:Homologous recombination | 2351573341 | 1622411425 | 0.17125 |
| ko00071:Fatty acid metabolism | 503751164 | 385248701.9 | 0.17247 |
| ko00240:Pyrimidine metabolism | 1836965235 | 1266188188 | 0.17247 |
| ko00362:Benzoate degradation | 232928876.6 | 181361678.1 | 0.17247 |
| ko03015:mRNA surveillance pathway | 80588.9219 | 145453.2609 | 0.17316 |
| ko00061:Fatty acid biosynthesis | 2464222210 | 1709107528 | 0.17369 |
| ko00230:Purine metabolism | 1376642987 | 970143590 | 0.17369 |
| ko00330:Arginine and proline metabolism | 1099748087 | 805566245.8 | 0.17369 |
| ko00620:Pyruvate metabolism | 1801090374 | 1273024645 | 0.17369 |
| ko03030:DNA replication | 1926637985 | 1329645715 | 0.17369 |
| ko04141:Protein processing in endoplasmic reticulum | 77966329.74 | 49853125.78 | 0.17369 |
| ko00020:Citrate cycle (TCA cycle) | 1700093750 | 1109098780 | 0.17492 |
| ko00140:Steroid hormone biosynthesis | 106176188.1 | 64373540.33 | 0.17492 |
| ko03010:Ribosome | 2478545865 | 1656142126 | 0.17492 |
| ko00190:Oxidative phosphorylation | 746192955.9 | 512513881.8 | 0.17616 |
| ko00564:Glycerophospholipid metabolism | 883599334.8 | 653658195 | 0.17616 |
| ko00640:Propanoate metabolism | 1030671188 | 717137668.2 | 0.17616 |
| ko01040:Biosynthesis of unsaturated fatty acids | 542869546.8 | 405014737.8 | 0.17616 |
| ko00312:beta-Lactam resistance | 348312360.9 | 322113795.7 | 0.17741 |
| ko05146:Amoebiasis | 22019326.26 | 15275648.66 | 0.17866 |
| ko00680:Methane metabolism | 799183024.1 | 599578711.6 | 0.17991 |
| ko04146:Peroxisome | 340029359.4 | 243560571.9 | 0.17991 |
| ko03420:Nucleotide excision repair | 1256093764 | 884582903.9 | 0.18118 |
| ko00300:Lysine biosynthesis | 2409302760 | 1707049049 | 0.18245 |
| ko00720:Carbon fixation pathways in prokaryotes | 1836018249 | 1205064519 | 0.18245 |
| ko04075:Plant hormone signal transduction | 383077.4248 | 238620.7604 | 0.18339 |
| ko00970:Aminoacyl-tRNA biosynthesis | 2556719054 | 1745360637 | 0.18372 |
| ko05012:Parkinson's disease | 1645835.486 | 2381429.735 | 0.18552 |
| ko00550:Peptidoglycan biosynthesis | 2880108253 | 1993378446 | 0.1863 |
| ko00630:Glyoxylate and dicarboxylate metabolism | 1023435552 | 744034720.3 | 0.1863 |
| ko00670:One carbon pool by folate | 2787661529 | 1912663121 | 0.1863 |
| ko02020:Two-component system | 483126926.6 | 390171569 | 0.1863 |
| ko03020:RNA polymerase | 1624973607 | 1137645710 | 0.1863 |
| ko03410:Base excision repair | 1430609305 | 991695679 | 0.1863 |
| ko01057:Biosynthesis of type II polyketide products | 49593.47464 | 4837.525641 | 0.18681 |
| ko00900:Terpenoid backbone biosynthesis | 2015940443 | 1376988445 | 0.18759 |
| ko03018:RNA degradation | 991173356.4 | 666315033.2 | 0.18759 |
| ko00660:C5-Branched dibasic acid metabolism | 2651359216 | 1934699796 | 0.1889 |
| ko04112:Cell cycle - Caulobacter | 2364713945 | 1628094018 | 0.1889 |
| ko04626:Plant-pathogen interaction | 281351540.9 | 204831396 | 0.1889 |
| ko04910:Insulin signaling pathway | 151624924.2 | 96612897.65 | 0.1889 |
| ko00260:Glycine, serine and threonine metabolism | 1659297696 | 1182863983 | 0.19152 |
| ko00400:Phenylalanine, tyrosine  and tryptophan biosynthesis | 1834059316 | 1278646053 | 0.19152 |
| ko01051:Biosynthesis of ansamycins | 6289135057 | 4891658282 | 0.19285 |
| ko00410:beta-Alanine metabolism | 465097368.5 | 428909895.8 | 0.19332 |
| ko00740:Riboflavin metabolism | 1377033144 | 902418086.9 | 0.19418 |
| ko00072:Synthesis and degradation of ketone bodies | 416561585.5 | 339334071.5 | 0.19551 |
| ko05111:Vibrio cholerae pathogenic cycle | 230208820.9 | 166234712.3 | 0.1982 |
| ko00750:Vitamin B6 metabolism | 1749049363 | 1229208310 | 0.19956 |
| ko03060:Protein export | 2225912481 | 1551606322 | 0.19956 |
| ko00290:Valine, leucine and isoleucine biosynthesis | 3295959744 | 2427595151 | 0.2023 |
| ko00920:Sulfur metabolism | 1229639339 | 864335751.6 | 0.2023 |
| ko01053:Biosynthesis of siderophore  group nonribosomal peptides | 116417388.5 | 113967203.2 | 0.2023 |
| ko05100:Bacterial invasion of epithelial cells | 9015557.295 | 14693261.32 | 0.20367 |
| ko00770:Pantothenate and CoA biosynthesis | 2739880916 | 1928783996 | 0.20645 |
| ko03008:Ribosome biogenesis in eukaryotes | 83851332.51 | 59763901.99 | 0.20785 |
| ko00941:Flavonoid biosynthesis | 16138733.63 | 8503105.428 | 0.21066 |
| ko00531:Glycosaminoglycan degradation | 1473583940 | 862655027.8 | 0.21494 |
| ko00785:Lipoic acid metabolism | 1948203607 | 1102589378 | 0.21494 |
| ko00621:Dioxin degradation | 277628540.6 | 278172615.1 | 0.22073 |
| ko03070:Bacterial secretion system | 1180526732 | 844644421.7 | 0.2222 |
| ko04080:Neuroactive ligand-receptor interaction | 0.488326087 | 6.814458974 | 0.22343 |
| ko00340:Histidine metabolism | 1791312323 | 1322859664 | 0.22515 |
| ko05150:Staphylococcus aureus infection | 46548249.81 | 36169230.72 | 0.23418 |
| ko04210:Apoptosis | 67986113.99 | 35270827.18 | 0.23725 |
| ko00130:Ubiquinone and other  terpenoid-quinone biosynthesis | 693960934.4 | 457938991.7 | 0.2388 |
| ko00906:Carotenoid biosynthesis | 22569937.42 | 10950985.93 | 0.2419 |
| ko00380:Tryptophan metabolism | 262969223.9 | 188404347.9 | 0.249 |
| ko04113:Meiosis - yeast | 416310.5885 | 331609.3768 | 0.26027 |
| ko02030:Bacterial chemotaxis | 1078600369 | 895439435 | 0.26116 |
| ko00930:Caprolactam degradation | 37552095.27 | 48988574.64 | 0.27627 |
| ko00965:Betalain biosynthesis | 53739.62391 | 11580.01058 | 0.27853 |
| ko00311:Penicillin and cephalosporin biosynthesis | 42390744.1 | 42804225.86 | 0.28318 |
| ko04974:Protein digestion and absorption | 89800171.81 | 51984028.22 | 0.29554 |
| ko00540:Lipopolysaccharide biosynthesis | 1348731988 | 800951064.7 | 0.30643 |
| ko00472:D-Arginine and D-ornithine metabolism | 113326512.4 | 123963417.3 | 0.32324 |
| ko00563:Glycosylphosphatidylinositol(GPI)- anchor biosynthesis | 0 | 7.104535897 | 0.35397 |
| ko02040:Flagellar assembly | 621484607 | 496281671.1 | 0.35865 |
| ko00514:Other types of O-glycan biosynthesis | 51688.86749 | 392963.5434 | 0.36859 |
| ko00524:Butirosin and neomycin biosynthesis | 160835042.2 | 88803125.6 | 0.37769 |
| ko05145:Toxoplasmosis | 155015.2362 | 57340.04828 | 0.38594 |
| ko04970:Salivary secretion | 311533.3319 | 98916.35684 | 0.38918 |
| ko00624:Polycyclic aromatic hydrocarbon degradation | 558216.3026 | 510492.7331 | 0.45406 |
| ko03050:Proteasome | 1507969.582 | 3455497.553 | 0.46202 |
| ko05143:African trypanosomiasis | 5908017.555 | 10021095.24 | 0.47391 |
| ko00523:Polyketide sugar unit biosynthesis | 161277204.9 | 116699657.8 | 0.47482 |
| ko04512:ECM-receptor interaction | 6682.108178 | 72.91758205 | 0.48562 |
| ko05144:Malaria | 2210.459561 | 18882.16873 | 0.50567 |
| ko04310:Wnt signaling pathway | 43.77166377 | 36.4912359 | 0.56546 |
| ko05130:Pathogenic Escherichia coli infection | 17682.74485 | 43559.65182 | 0.5739 |
| ko04020:Calcium signaling pathway | 58474.66232 | 1112.686081 | 0.57847 |
| ko00281:Geraniol degradation | 142997103.2 | 107431851.5 | 0.59352 |
| ko00903:Limonene and pinene degradation | 65290010.58 | 53385578.32 | 0.60355 |
| ko00626:Naphthalene degradation | 115004619.3 | 104001222.9 | 0.64159 |
| ko00601:Glycosphingolipid biosynthesis -  lacto and neolacto series | 549674.2038 | 240280.3795 | 0.64331 |
| ko00623:Toluene degradation | 39429030.12 | 113702346.1 | 0.64503 |
| ko00943:Isoflavonoid biosynthesis | 31772.1646 | 14233.49771 | 0.64602 |
| ko00642:Ethylbenzene degradation | 54108750.17 | 46655220.41 | 0.67717 |
| ko04142:Lysosome | 359966.6708 | 28584475.52 | 0.72215 |
| ko00830:Retinol metabolism | 62704861.2 | 40084933.85 | 0.72292 |
| ko05322:Systemic lupus erythematosus | 52880.04064 | 64086.19723 | 0.7523 |
| ko00363:Bisphenol degradation | 112324909.9 | 30208482.45 | 0.77244 |
| ko05142:Chagas disease (American trypanosomiasis) | 209461.8508 | 60874.1782 | 0.77619 |
| ko00195:Photosynthesis | 110609798 | 75263792.17 | 0.7945 |
| ko00591:Linoleic acid metabolism | 219742784 | 181300193.7 | 0.80322 |
| ko00625:Chloroalkane and chloroalkene degradation | 281900918.1 | 208228830.7 | 0.83644 |
| ko04110:Cell cycle | 1541.304904 | 20381.02465 | 0.84382 |
| ko00364:Fluorobenzoate degradation | 12252802.29 | 17736132.83 | 0.88665 |
| ko00622:Xylene degradation | 60153244.5 | 50630797.94 | 0.88833 |
| ko00460:Cyanoamino acid metabolism | 265179670.6 | 274692024 | 0.89155 |
| ko00643:Styrene degradation | 52317746.66 | 48347669.26 | 0.89213 |
| ko00196:Photosynthesis - antenna proteins | 37978.19462 | 58757.85383 | 0.92674 |
| ko00983:Drug metabolism - other enzymes | 450284240 | 169850558.3 | 0.93815 |
| ko00513:Various types of N-glycan biosynthesis | 367.84 | 3546.573077 | 0.94602 |
| ko00980:Metabolism of xenobiotics  by cytochrome P450 | 10956301.35 | 17894733.82 | 0.99371 |

Footnote: KEGG_Pathway: KEGG pathway; Mean in NLNM: the predicted abundance value of this pathway in each sample in NLNM; Mean in LNM: the predicted abundance value of this pathway in each sample in LNM.
