# Supplementary material for: Identification and predictive machine learning models construction of gut microbiota associated with lymph node metastasis in colorectal cancer
Source: mSystems. 2025 Jul 8;10(8):e00339-25. doi: 10.1128/msystems.00339-25 (PMC12363233; doi:10.1128/msystems.00339-25)
Supplement: Table S3 — List of differential GO items and KEGG pathways of LNM and NLNM. [file msystems.00339-25-s0006.pdf]

Table.S3. List of differential GO items of LNM

GO items in LNM	logFC	P.Value
GOBP_ACTIN_MYOSIN_FILAMENT_SLIDING	0.033	0.0007
GOBP_LEUKOTRIENE_BIOSYNTHETIC_PROCESS	0.038	0.0009
GOBP_REGULATION_OF_SYNAPSE_MATURATION	0.023	0.001
GOCC_EXTRINSIC_COMPONENT_OF_ORGANELLE_MEMBRANE	0.012	0.0013
GOCC_MUSCLE_MYOSIN_COMPLEX	0.033	0.002
GOMF_IONOTROPIC_GLUTAMATE_RECEPTOR_ACTIVITY	0.023	0.0034
GOBP_MEMBRANE_DISASSEMBLY	0.021	0.0043
GOBP_CELLULAR_RESPONSE_TO_ESTROGEN_STIMULUS	0.016	0.0043
GOBP_HISTONE_H3_K27_METHYLATION	0.021	0.0048
GOBP_LIGAND_GATED_ION_CHANNEL_SIGNALING_PATHWAY	0.017	0.0055
GOMF_LRR_DOMAIN_BINDING	0.016	0.0063
GOBP_URINARY_TRACT_SMOOTH_MUSCLE_CONTRACTION	0.05	0.0072
GOBP_MAINTENANCE_OF_CELL_POLARITY	0.017	0.0075
GOMF_THREONINE_TYPE_PEPTIDASE_ACTIVITY	0.025	0.0102
GOCC_PHOTORECEPTOR_DISC_MEMBRANE	0.013	0.0122
GOBP_LEUKOTRIENE_D4_METABOLIC_PROCESS	0.052	0.0126
GOMF_STRUCTURAL_CONSTITUENT_OF_EYE_LENS	0.031	0.013
GOBP_REGULATION_OF_PROTEIN_COMPLEX_STABILITY	0.02	0.0132
GOCC_POST_MRNA_RELEASE_SPLICEOSOMAL_COMPLEX	0.021	0.0136
GOBP_ACTIN_CROSSLINK_FORMATION	0.018	0.0149
GOMF_EXTRACELLULAR_LIGAND_GATED_ION_CHANNEL_ACTIVITY	0.013	0.0174
GOBP_REGULATION_OF_ORGANELLE_TRANSPORT _ALONG_MICROTUBULE	0.026	0.0176
GOCC_VACUOLAR_PROTON_TRANSPORTING_V _TYPE_ATPASE_V0_DOMAIN	0.013	0.0194
GOBP_POSITIVE_REGULATION_OF_EXCITATORY _POSTSYNAPTIC_POTENTIAL	0.021	0.0198
GOBP_PHOSPHATIDYLCHOLINE_ACYL_CHAIN_REMODELING	0.021	0.0209
GOCC_CYTOPLASMIC_SIDE_OF_LYSOSOMAL_MEMBRANE	0.024	0.0219
GOMF_HORMONE_ACTIVITY	0.018	0.0229
GOMF_OMEGA_PEPTIDASE_ACTIVITY	0.039	0.0239
GOBP_POSITIVE_REGULATION_OF_MITOCHONDRIAL _MEMBRANE_POTENTIAL	0.014	0.0241
GOBP_NOREPINEPHRINE_SECRETION	0.034	0.0251
GOBP_POSITIVE_REGULATION_OF_CILIUM_MOVEMENT	0.026	0.0259
GOBP_CYTIDINE_METABOLIC_PROCESS	0.038	0.0264
GOMF_CYTIDINE_DEAMINASE_ACTIVITY	0.038	0.0264
GOMF_AMINOACYLTRANSFERASE_ACTIVITY	0.032	0.0268
GOBP_REGULATION_OF_RELEASE_OF_CYTOCHROME _C_FROM_MITOCHONDRIA	0.013	0.0269
GOMF_CHLORIDE_ION_BINDING	0.024	0.0271
GOBP_REGULATION_OF_POSTSYNAPTIC_DENSITY_ORGANIZATION	0.022	0.0275
GOMF_MYOSIN_HEAVY_CHAIN_BINDING	0.036	0.0275
GOCC_CATSPER_COMPLEX	0.018	0.028
GOBP_LONG_CHAIN_FATTY_ACID_BIOSYNTHETIC_PROCESS	0.021	0.0284
GOMF_NEUROPEPTIDE_HORMONE_ACTIVITY	0.036	0.0285
GOCC_PROTON_TRANSPORTING_V_TYPE_ATPASE_V0_DOMAIN	0.011	0.0285
GOMF_PHOSPHOLIPASE_A2_ACTIVITY	0.019	0.0289
GOMF_CALCIUM_ACTIVATED_CATION_CHANNEL_ACTIVITY	0.017	0.0298
GOBP_SYNAPSE_MATURATION	0.021	0.0299
GOCC_MYOSIN_II_COMPLEX	0.015	0.0301
GOBP_CYTOLYSIS	0.038	0.0309
GOCC_KINOCILIUM	0.018	0.0321
GOBP_LEUKOTRIENE_METABOLIC_PROCESS	0.022	0.0322
GOMF_RDNA_BINDING	0.021	0.0337
GOBP_PHOSPHATIDYLGLYCEROL_BIOSYNTHETIC_PROCESS	0.032	0.0338

GOBP_REGULATION_OF_FLAGELLATED_SPERM_MOTILITY	0.023	0.0343
GOBP_IRON_COORDINATION_ENTITY_TRANSPORT	0.019	0.0354
GOBP_POSITIVE_REGULATION_OF_SIGNAL_TRANSDUCTION_BY _P53_CLASS_MEDIATOR	0.011	0.0354
GOBP_REGULATION_OF_CELL_MATURATION	0.014	0.0392
GOBP_ICOSANOID_BIOSYNTHETIC_PROCESS	0.014	0.0402
GOBP_REGULATION_OF_ACUTE_INFLAMMATORY_RESPONSE _TO_ANTIGENIC_STIMULUS	0.028	0.0412
GOMF_FERROUS_IRON_BINDING	0.011	0.0424
GOBP_NEGATIVE_REGULATION_OF_INTERLEUKIN_17_PRODUCTION	0.021	0.0432
GOBP_MUSCLE_CELL_FATE_COMMITMENT	0.025	0.0443
GOBP_INOSITOL_TRISPHOSPHATE_METABOLIC_PROCESS	0.033	0.0443
GOBP_DEFENSE_RESPONSE_TO_FUNGUS	0.019	0.0466
GOBP_REGULATION_OF_PHOSPHOLIPASE_A2_ACTIVITY	0.023	0.0484
GOMF_CHONDROITIN_SULFATE_BINDING	0.033	0.0487
GOBP_NOREPINEPHRINE_TRANSPORT	0.029	0.0493

Footnote: GO items: Enriched GO entries. LogFC: FC represents the folding change, that is, the ratio of

Table.S3. List of differential GO items of NLNM

GO items in NLNM	logFC	P.Value
GOBP_ESTABLISHMENT_OF_ENDOTHELIAL_INTESTINAL_BARRIER	0.048	0.0001
GOBP_ER_NUCLEUS_SIGNALING_PATHWAY	0.022	0.0002
GOBP_NUCLEAR_PORE_COMPLEX_ASSEMBLY	0.054	0.0003
GOCC_FILOPODIUM_TIP	0.027	0.0004
GOBP_PORE_COMPLEX_ASSEMBLY	0.03	0.0005
GOBP_LONG_CHAIN_FATTY_ACYL_COA_METABOLIC_PROCESS	0.034	0.0005
GOBP_ACTIVATION_OF_NF_KAPPAB_INDUCING_KINASE_ACTIVITY	0.023	0.0005
GOBP_LOW_DENSITY_LIPOPROTEIN_PARTICLE_CLEARANCE	0.023	0.0006
GOBP_ENDOPLASMIC_RETICULUM_UNFOLDED_PROTEIN_RESPONSE	0.019	0.0007
GOBP_CELLULAR_RESPONSE_TO_UNFOLDED_PROTEIN	0.016	0.0008
GOBP_CELLULAR_RESPONSE_TO_TOPOLOGICALLY_INCORRECT_PROTE	0.015	0.0008
GOBP_REGULATION_OF_IRE1_MEDIATED_UNFOLDED _PROTEIN_RESPONSE	0.029	0.0009
GOBP_ER_OVERLOAD_RESPONSE	0.028	0.0009
GOBP_HEPARAN_SULFATE_PROTEOGLYCAN_BIOSYNTHETIC_PROCESS	0.022	0.001
GOMF_PHOSPHOLIPID_SCRAMBLASE_ACTIVITY	0.018	0.0011
GOBP_REGULATION_OF_ENDOPLASMIC_RETICULUM _UNFOLDED_PROTEIN_RESPONSE	0.018	0.0012
GOMF_LIPOPROTEIN_LIPASE_ACTIVITY	0.035	0.0012
GOBP_PURINE_NUCLEOSIDE_MONOPHOSPHATE_METABOLIC_PROCESS	0.026	0.0013
GOBP_LONG_CHAIN_FATTY_ACYL_COA_BIOSYNTHETIC_PROCESS	0.031	0.0014
GOCC_NUCLEAR_INCLUSION_BODY	0.031	0.0014
GOBP_VESICLE_TARGETING	0.015	0.0015
GOBP_NUCLEAR_PORE_ORGANIZATION	0.043	0.0015
GOMF_PHOSPHATIDYLINOSITOL_3_5_BISPHOSPHATE _3_PHOSPHATASE_ACTIVITY	0.047	0.0017
GOBP_REGULATION_OF_TRANSLATIONAL_INITIATION_BY_EIF2 _ALPHA_PHOSPHORYLATION	0.043	0.0017
GOBP_N_TERMINAL_PROTEIN_AMINO_ACID_MODIFICATION	0.017	0.0018
GOBP_REGULATION_OF_TRANSLATIONAL_INITIATION _IN_RESPONSE_TO_STRESS	0.033	0.0018
GOBP_RESPONSE_TO_STEROL_DEPLETION	0.028	0.002
GOMF_NUCLEOTIDE_SUGAR_TRANSMEMBRANE _TRANSPORTER_ACTIVITY	0.047	0.002
GOMF_POLY_A_SPECIFIC_RIBONUCLEASE_ACTIVITY	0.033	0.002
GOMF_ANTIPORTER_ACTIVITY	0.018	0.0021
GOBP_N_TERMINAL_PROTEIN_AMINO_ACID_ACETYLATION	0.02	0.0022
GOBP_RIBONUCLEOSIDE_MONOPHOSPHATE_METABOLIC_PROCESS	0.023	0.0022

GOBP_NEGATIVE_REGULATION_OF_ENDOPLASMIC_RETICULUM _UNFOLDED_PROTEIN_RESPONSE	0.023	0.0023
GOBP_REGULATION_OF_ENDOPLASMIC_RETICULUM_STRESS _INDUCED_INTRINSIC_APOPTOTIC_SIGNALING_PATHWAY	0.013	0.0023
GOBP_LONG_CHAIN_FATTY_ACID_IMPORT_ACROSS _PLASMA_MEMBRANE	0.026	0.0024
GOCC_EXTRINSIC_COMPONENT_OF_SYNAPTIC_MEMBRANE	0.021	0.0024
GOBP_RESPONSE_TO_FATTY_ACID	0.018	0.0026
GOBP_TRANSEPITHELIAL_TRANSPORT	0.029	0.0026
GOBP_PYRIMIDINE_RIBONUCLEOTIDE_METABOLIC_PROCESS	0.017	0.0027
GOBP_RESPONSE_TO_LEAD_ION	0.03	0.0027
GOMF_PYRIMIDINE_NUCLEOTIDE_SUGAR_TRANSMEMBRANE _TRANSPORTER_ACTIVITY	0.048	0.0028
GOMF_ALPHA_CATENIN_BINDING	0.04	0.003
GOBP_FATTY_ACYL_COA_BIOSYNTHETIC_PROCESS	0.021	0.003
GOBP_MRNA_TRANSCRIPTION	0.02	0.0031
GOBP_REGULATION_OF_CHOLESTEROL_BIOSYNTHETIC_PROCESS	0.02	0.0033
GOBP_IRE1_MEDIATED_UNFOLDED_PROTEIN_RESPONSE	0.026	0.0034
GOBP_RECEPTOR_CATABOLIC_PROCESS	0.017	0.0034
GOBP_PROTEIN_K48_LINKED_UBIQUITINATION	0.017	0.0034
GOBP_AMINO_ACID_IMPORT_ACROSS_PLASMA_MEMBRANE	0.021	0.0035
GOMF_CARBOHYDRATE_TRANSMEMBRANE_TRANSPORTER_ACTIVITY	0.021	0.0035
GOBP_MAMMARY_GLAND_EPITHELIAL_CELL_PROLIFERATION	0.019	0.0035
GOBP_POSITIVE_REGULATION_BY_HOST_OF_VIRAL_TRANSCRIPTION	0.031	0.0036
GOBP_CYTOKINESIS	0.013	0.0036
GOBP_HEPATICOBILIARY_SYSTEM_DEVELOPMENT	0.019	0.0038
GOMF_PHOSPHATIDYLINOSITOL_3_5_BISPHOSPHATE _PHOSPHATASE_ACTIVITY	0.041	0.0038
GOBP_MAMMARY_GLAND_ALVEOLUS_DEVELOPMENT	0.035	0.0038
GOCC_APICOLATERAL_PLASMA_MEMBRANE	0.029	0.0039
GOMF_SULFUR_AMINO_ACID_TRANSMEMBRANE _TRANSPORTER_ACTIVITY	0.031	0.0042
GOBP_POSITIVE_REGULATION_OF_TELOMERE_MAINTENANCE _VIA_TELOMERE_LENGTHENING	0.017	0.0042
GOMF_CADHERIN_BINDING	0.016	0.0044
GOBP_REGULATION_OF_TRANSLATION_IN_RESPONSE_TO_STRESS	0.03	0.0045
GOBP_NUCLEOSIDE_BISPHOSPHATE_BIOSYNTHETIC_PROCESS	0.02	0.0046
GOBP_POSITIVE_REGULATION_OF_POST _TRANSCRIPTIONAL_GENE_SILENCING	0.027	0.0046
GOBP_POSITIVE_REGULATION_OF_RESPONSE_TO _ENDOPLASMIC_RETICULUM_STRESS	0.012	0.0046
GOBP_NADPH_REGENERATION	0.019	0.0048
GOBP_RESPONSE_TO_EPIDERMAL_GROWTH_FACTOR	0.021	0.0048
GOMF_RNA_POLYMERASE_BINDING	0.017	0.0048
GOBP_RESPONSE_TO_TOPOLOGICALLY_INCORRECT_PROTEIN	0.013	0.0049
GOCC_MESSENGER_RIBONUCLEOPROTEIN_COMPLEX	0.022	0.005
GOBP_NEGATIVE_REGULATION_OF_RESPONSE_TO_OXIDATIVE_STRESS	0.018	0.005
GOBP_NEURON_DEATH_IN_RESPONSE_TO_OXIDATIVE_STRESS	0.019	0.005
GOBP_THIOESTER_BIOSYNTHETIC_PROCESS	0.019	0.0054
GOBP_CELLULAR_RESPONSE_TO_STEROL_DEPLETION	0.026	0.0055
GOBP_REGULATION_OF_TRANSLATION_IN_RESPONSE _TO_ENDOPLASMIC_RETICULUM_STRESS	0.04	0.0055
GOMF_MODIFIED_AMINO_ACID_TRANSMEMBRANE _TRANSPORTER_ACTIVITY	0.029	0.0057
GOCC_RNA_CAP_BINDING_COMPLEX	0.019	0.0059
GOBP_NLS_BEARING_PROTEIN_IMPORT_INTO_NUCLEUS	0.023	0.006

GOBP_NEGATIVE_REGULATION_OF_NUCLEAR _TRANSCRIBED_MRNA_CATABOLIC_PROCESS _DEADENYLATION_DEPENDENT_DECAY	0.019	0.0062
GOBP_PLATELET_MORPHOGENESIS	0.021	0.0064
GOMF_PHOSPHOTRANSFERASE_ACTIVITY_FOR _OTHER_SUBSTITUTED_PHOSPHATE_GROUPS	0.022	0.0065
GOBP_PEPTIDYL_THREONINE_MODIFICATION	0.015	0.0065
GOBP_REGULATION_OF_SECONDARY_METABOLIC_PROCESS	0.02	0.0066
GOCC_RNA_POLYMERASE_II_TRANSCRIPTION_REPRESSOR_COMPLEX	0.019	0.0066
GOBP_HYPOTONIC_RESPONSE	0.039	0.0068
GOMF_RIBOSOMAL_SMALL_SUBUNIT_BINDING	0.017	0.0068
GOBP_HEPARAN_SULFATE_PROTEOGLYCAN_METABOLIC_PROCESS	0.021	0.007
GOBP_NUCLEOTIDE_SUGAR_TRANSMEMBRANE_TRANSPORT	0.043	0.007
GOBP_POSITIVE_REGULATION_OF_RNA_SPLICING	0.015	0.007
GOMF_VOLTAGE_GATED_CHLORIDE_CHANNEL_ACTIVITY	0.028	0.0071
GOBP_TELOMERE_CAPPING	0.016	0.0071
GOMF_MDM2_MDM4_FAMILY_PROTEIN_BINDING	0.023	0.0072
GOBP_L_ASPARTATE_TRANSMEMBRANE_TRANSPORT	0.026	0.0072
GOBP_BERGMANN_GLIAL_CELL_DIFFERENTIATION	0.034	0.0073
GOBP_FATTY_ACYL_COA_METABOLIC_PROCESS	0.021	0.0073
GOMF_VOLTAGE_GATED_ANION_CHANNEL_ACTIVITY	0.018	0.0073
GOBP_POSITIVE_REGULATION_OF_MRNA_PROCESSING	0.016	0.0074
GOBP_GLUCOSE_6_PHOSPHATE_METABOLIC_PROCESS	0.021	0.0074
GOBP_MANGANESE_ION_TRANSPORT	0.029	0.0074
GOMF_CYCLIN_DEPENDENT_PROTEIN_SERINE _THREONINE_KINASE_ACTIVATOR_ACTIVITY	0.03	0.0076
GOBP_REGULATION_OF_RNA_POLYMERASE_II _REGULATORY_REGION_SEQUENCE_SPECIFIC_DNA_BINDING	0.026	0.0076
GOBP_HEPATOCYTE_APOPTOTIC_PROCESS	0.023	0.0076
GOBP_PRIMARY_MIRNA_PROCESSING	0.023	0.0077
GOBP_OTOLITH_DEVELOPMENT	0.027	0.008
GOBP_NEGATIVE_REGULATION_OF_CIRCADIAN_RHYTHM	0.025	0.008
GOBP_NEGATIVE_REGULATION_OF_MYELOID_CELL _APOPTOTIC_PROCESS	0.02	0.008
GOBP_GMP_METABOLIC_PROCESS	0.023	0.0081
GOBP_POSITIVE_REGULATION_OF_MRNA_3_END_PROCESSING	0.023	0.0082
GOBP_THIOESTER_METABOLIC_PROCESS	0.015	0.0084
GOMF_ATP_DEPENDENT_ACTIVITY	0.012	0.0085
GOCC_KINETOCHORE_MICROTUBULE	0.015	0.0087
GOBP_ENDOPLASMIC_RETICULUM_MANNOSE_TRIMMING	0.025	0.0088
GOBP_REGULATION_OF_OXIDATIVE_STRESS_INDUCED_CELL_DEATH	0.016	0.0088
GOCC_ORGANELLE_MEMBRANE_CONTACT_SITE	0.014	0.0089
GOMF_SIGNAL_SEQUENCE_BINDING	0.017	0.0089
GOMF_H4_HISTONE_ACETYLTRANSFERASE_ACTIVITY	0.027	0.009
GOBP_NEGATIVE_REGULATION_OF_KERATINOCYTE_PROLIFERATION	0.021	0.0091
GOBP_NUCLEUS_ORGANIZATION	0.013	0.0091
GOMF_ADENYL_NUCLEOTIDE_BINDING	0.01	0.0092
GOBP_POSITIVE_REGULATION_OF_ANION _TRANSMEMBRANE_TRANSPORT	0.043	0.0093
GOBP_REGULATION_OF_CLATHRIN_DEPENDENT_ENDOCYTOSIS	0.029	0.0093
GOBP_POSTSYNAPTIC_MODULATION_OF _CHEMICAL_SYNAPTIC_TRANSMISSION	0.023	0.0095
GOBP_NEGATIVE_REGULATION_OF_HISTONE_H3_K9_METHYLATION	0.02	0.0097
GOBP_REGULATION_OF_CYTOKINESIS	0.019	0.0098
GOBP_REGULATION_OF_TRANSCRIPTION_INVOLVED _IN_G1_S_TRANSITION_OF_MITOTIC_CELL_CYCLE	0.019	0.0098
GOBP_SERTOLI_CELL_DEVELOPMENT	0.023	0.0098
GOMF_KINASE_ACTIVATOR_ACTIVITY	0.012	0.0098

GOBP_LABYRINTHINE_LAYER_DEVELOPMENT	0.018	0.0099
GOMF_POLYOL_TRANSMEMBRANE_TRANSPORTER_ACTIVITY	0.037	0.01
GOBP_HISTONE_H3_K36_DEMETHYLATION	0.025	0.0101
GOMF_HISTONE_H3_METHYL_LYSINE_36_DEMETHYLASE_ACTIVITY	0.025	0.0101
GOMF_ATP_HYDROLYSIS_ACTIVITY	0.013	0.0101
GOCC_ENDOPLASMIC_RETICULUM_QUALITY_CONTROL_COMPARTMEN	0.023	0.0102
GOBP_REGULATION_OF_PROTEIN_CATABOLIC _PROCESS_IN_THE_VACUOLE	0.022	0.0104
GOMF_PHOSPHATIDYLINOSITOL_MONOPHOSPHATE _PHOSPHATASE_ACTIVITY	0.03	0.0104
GOBP_POSITIVE_REGULATION_OF_HISTONE_H3_K4_METHYLATION	0.019	0.0104
GOCC_SEX_CHROMOSOME	0.016	0.0104
GOBP_NEGATIVE_REGULATION_OF_GLUCONEOGENESIS	0.02	0.0106
GOBP_POSITIVE_REGULATION_OF_NEURON_MIGRATION	0.023	0.0107
GOBP_GASTRULATION_WITH_MOUTH_FORMING_SECOND	0.024	0.0108
GOMF_MONOVALENT_CATION_PROTON_ANTIPORTER_ACTIVITY	0.026	0.0109
GOBP_AMP_METABOLIC_PROCESS	0.025	0.011
GOMF_ALANINE_TRANSMEMBRANE_TRANSPORTER_ACTIVITY	0.031	0.011
GOBP_RESPONSE_TO_NITROSATIVE_STRESS	0.022	0.011
GOBP_NUCLEOTIDE_TRANSMEMBRANE_TRANSPORT	0.019	0.0111
GOMF_PROTEIN_SERINE_THREONINE_KINASE_ACTIVATOR_ACTIVITY	0.019	0.0111
GOBP_PROTEIN_LOCALIZATION_TO_LYSOSOME	0.016	0.0112
GOBP_NEUTROPHIL_DIFFERENTIATION	0.023	0.0112
GOBP_RETINA_MORPHOGENESIS_IN_CAMERA_TYPE_EYE	0.016	0.0115
GOBP_ESTABLISHMENT_OF_EPITHELIAL_CELL_POLARITY	0.017	0.0115
GOMF_ACIDIC_AMINO_ACID_TRANSMEMBRANE _TRANSPORTER_ACTIVITY	0.021	0.0117
GOMF_PRE_MRNA_INTRONIC_BINDING	0.029	0.0117
GOBP_REGULATION_OF_TOLL_LIKE_RECEPTOR_3_SIGNALING_PATHWA	0.037	0.0118
GOMF_DIPEPTIDYL_PEPTIDASE_ACTIVITY	0.021	0.0118
GOBP_NUCLEAR_TRANSCRIBED_MRNA_POLY_A_TAIL_SHORTENING	0.019	0.012
GOBP_PROTEIN_QUALITY_CONTROL_FOR_MISFOLDED_OR _INCOMPLETELY_SYNTHESIZED_PROTEINS	0.012	0.012
GOBP_C4_DICARBOXYLATE_TRANSPORT	0.022	0.012
GOBP_ORGANELLE_INHERITANCE	0.015	0.012
GOBP_HISTONE_MONOUBIQUITINATION	0.021	0.0121
GOBP_POSITIVE_REGULATION_OF_PEPTIDYL_LYSINE_ACETYLATION	0.013	0.0121
GOCC_NUCLEAR_CYCLIN_DEPENDENT_PROTEIN_KINASE _HOLOENZYME_COMPLEX	0.025	0.0122
GOCC_GOLGI_STACK	0.017	0.0125
GOMF_NUCLEOSIDE_MONOPHOSPHATE_KINASE_ACTIVITY	0.021	0.0127
GOBP_NEGATIVE_REGULATION_OF_HOMOTYPIC_CELL_CELL_ADHESIO	0.027	0.0129
GOCC_PIGMENT_GRANULE	0.011	0.0129
GOCC_NUCLEAR_MEMBRANE	0.01	0.013
GOBP_CELL_DIFFERENTIATION_INVOLVED_IN_EMBRYONIC _PLACENTA_DEVELOPMENT	0.024	0.013
GOBP_GLUCOSE_METABOLIC_PROCESS	0.012	0.013
GOBP_RESPONSE_TO_GROWTH_HORMONE	0.018	0.013
GOBP_REGULATION_OF_KERATINOCYTE_PROLIFERATION	0.019	0.0131
GOBP_TRICARBOXYLIC_ACID_CYCLE	0.018	0.0133
GOMF_ARACHIDONATE_COA_LIGASE_ACTIVITY	0.021	0.0133
GOMF_HEPARAN_SULFATE_SULFOTRANSFERASE_ACTIVITY	0.02	0.0134
GOCC_CUL4_RING_E3_UBIQUITIN_LIGASE_COMPLEX	0.013	0.0136
GOBP_MIRNA_CATABOLIC_PROCESS	0.018	0.0139
GOCC_CCR4_NOT_COMPLEX	0.018	0.0139
GOBP_PROTEIN_LOCALIZATION_TO_MICROTUBULE _ORGANIZING_CENTER	0.014	0.0141
GOBP_HISTONE_UBIQUITINATION	0.02	0.0141

GOMF_SULFUR_COMPOUND_TRANSMEMBRANE _TRANSPORTER_ACTIVITY	0.024	0.0141
GOBP_REGULATION_OF_GLIAL_CELL_PROLIFERATION	0.017	0.0141
GOBP_RESPONSE_TO_DEXAMETHASONE	0.016	0.0143
GOMF_NUCLEAR_VITAMIN_D_RECEPTOR_BINDING	0.02	0.0143
GOBP_PROGRAMMED_CELL_DEATH_IN_RESPONSE _TO_REACTIVE_OXYGEN_SPECIES	0.012	0.0143
GOBP_VESICLE_TARGETING_TO_FROM_OR_WITHIN_GOLGI	0.013	0.0146
GOBP_POSITIVE_REGULATION_OF_AMINO_ACID_TRANSPORT	0.024	0.015
GOBP_ESTABLISHMENT_OF_SISTER_CHROMATID_COHESION	0.028	0.015
GOBP_CELLULAR_AMINO_ACID_BIOSYNTHETIC_PROCESS	0.013	0.0155
GOCC_BASAL_PART_OF_CELL	0.011	0.0157
GOBP_DNA_DOUBLE_STRAND_BREAK_PROCESSING	0.019	0.0157
GOBP_RESPONSE_TO_ENDOPLASMIC_RETICULUM_STRESS	0.01	0.0159
GOMF_UBIQUITIN_SPECIFIC_PROTEASE_BINDING	0.013	0.016
GOCC_DESMOSOME	0.033	0.016
GOBP_GLUTAMINE_FAMILY_AMINO_ACID_BIOSYNTHETIC_PROCESS	0.019	0.0161
GOMF_PRIMARY_MIRNA_BINDING	0.028	0.0162
GOBP_MITOTIC_SISTER_CHROMATID_COHESION	0.022	0.0162
GOBP_MAMMARY_GLAND_DEVELOPMENT	0.012	0.0165
GOBP_MONOUBIQUITINATED_PROTEIN_DEUBIQUITINATION	0.014	0.0165
GOBP_EPITHELIAL_FLUID_TRANSPORT	0.042	0.0165
GOBP_POSITIVE_REGULATION_OF_ENDOPLASMIC_RETICULUM _UNFOLDED_PROTEIN_RESPONSE	0.021	0.0165
GOBP_CARBOHYDRATE_DERIVATIVE_BIOSYNTHETIC_PROCESS	0.011	0.0166
GOBP_TRANSPORT_OF_VIRUS	0.018	0.0167
GOBP_POSITIVE_REGULATION_OF_MESENCHYMAL _CELL_PROLIFERATION	0.023	0.0167
GOBP_PROTEIN_CATABOLIC_PROCESS_IN_THE_VACUOLE	0.018	0.0167
GOBP_2_OXOGLUTARATE_METABOLIC_PROCESS	0.017	0.0168
GOBP_MAINTENANCE_OF_SISTER_CHROMATID_COHESION	0.017	0.0168
GOBP_PROTEIN_O_LINKED_GLYCOSYLATION	0.019	0.0169
GOBP_REGULATION_OF_TOLL_LIKE_RECEPTOR _2_SIGNALING_PATHWAY	0.028	0.017
GOBP_PHOSPHATIDYLSERINE_METABOLIC_PROCESS	0.024	0.0173
GOCC_CUL4A_RING_E3_UBIQUITIN_LIGASE_COMPLEX	0.017	0.0174
GOBP_HISTONE_H2A_MONOUBIQUITINATION	0.018	0.0175
GOBP_ERBB2_SIGNALING_PATHWAY	0.037	0.0176
GOBP_LACTATION	0.017	0.0177
GOBP_IMPORT_INTO_NUCLEUS	0.011	0.0177
GOBP_PROTEIN_LOCALIZATION_TO_VACUOLE	0.015	0.0178
GOBP_BRANCH_ELONGATION_OF_AN_EPITHELIUM	0.025	0.0179
GOCC_CORTICAL_ACTIN_CYTOSKELETON	0.015	0.018
GOBP_REGULATION_OF_GLUCOSE_METABOLIC_PROCESS	0.013	0.018
GOBP_XENOBIOTIC_TRANSPORT	0.021	0.018
GOCC_MLL3_4_COMPLEX	0.021	0.0181
GOMF_RNA_POLYMERASE_CORE_ENZYME_BINDING	0.014	0.0181
GOBP_NEGATIVE_REGULATION_OF_RESPONSE_TO _REACTIVE_OXYGEN_SPECIES	0.013	0.0182
GOMF_XENOBIOTIC_TRANSMEMBRANE_TRANSPORTER_ACTIVITY	0.02	0.0182
GOMF_TRANSMEMBRANE_RECEPTOR_PROTEIN_TYROSINE _KINASE_ACTIVATOR_ACTIVITY	0.038	0.0184
GOBP_RECEPTOR_METABOLIC_PROCESS	0.014	0.0184
GOBP_SULFUR_COMPOUND_TRANSPORT	0.021	0.0184
GOBP_POSITIVE_REGULATION_OF_TELOMERASE_ACTIVITY	0.017	0.0185
GOBP_ENDOPLASMIC_RETICULUM_TO_GOLGI_VESICLE _MEDIATED_TRANSPORT	0.012	0.0186
GOBP_REFLEX	0.02	0.0186

GOBP_RESPONSE_TO_FORSKOLIN	0.03	0.0186
GOBP_HISTONE_H2A_UBIQUITINATION	0.018	0.0187
GOBP_PHOSPHORYLATION_OF_RNA_POLYMERASE_II _C_TERMINAL_DOMAIN	0.022	0.019
GOBP_NEGATIVE_REGULATION_OF_STEROID_METABOLIC_PROCESS	0.02	0.0193
GOBP_RESPONSE_TO_MANGANESE_ION	0.019	0.0193
GOBP_POSITIVE_REGULATION_OF_STRIATED_MUSCLE _CELL_APOPTOTIC_PROCESS	0.023	0.0196
GOBP_PROTEIN_MODIFICATION_BY_SMALL_PROTEIN_REMOVAL	0.012	0.0199
GOMF_ATP_DEPENDENT_CHROMATIN_REMODELER_ACTIVITY	0.022	0.02
GOMF_NUCLEAR_LOCALIZATION_SEQUENCE_BINDING	0.023	0.0202
GOBP_REGULATION_OF_RECEPTOR_CATABOLIC_PROCESS	0.019	0.0202
GOBP_PROTEIN_K63_LINKED_DEUBIQUITINATION	0.013	0.0203
GOBP_CELLULAR_RESPONSE_TO_DEXAMETHASONE_STIMULUS	0.021	0.0204
GOBP_NEGATIVE_REGULATION_OF_ENDOPLASMIC_RETICULUM _STRESS_INDUCED_INTRINSIC_APOPTOTIC_SIGNALING_PATHWAY	0.013	0.0204
GOBP_LABYRINTHINE_LAYER_MORPHOGENESIS	0.024	0.0208
GOBP_POSITIVE_REGULATION_OF_MIRNA_MATURATION	0.022	0.0209
GOMF_NUCLEAR_THYROID_HORMONE_RECEPTOR_BINDING	0.018	0.021
GOBP_PHOSPHATIDYLINOSITOL_BIOSYNTHETIC_PROCESS	0.013	0.021
GOBP_POSITIVE_REGULATION_OF_PROTEIN_EXIT_FROM _ENDOPLASMIC_RETICULUM	0.035	0.0211
GOCC_BASOLATERAL_PLASMA_MEMBRANE	0.012	0.0212
GOBP_EMBRYONIC_PLACENTA_DEVELOPMENT	0.015	0.0213
GOMF_HELICASE_ACTIVITY	0.016	0.0216
GOBP_INTRACELLULAR_MRNA_LOCALIZATION	0.022	0.0216
GOMF_TRANSITION_METAL_ION_TRANSMEMBRANE _TRANSPORTER_ACTIVITY	0.024	0.0217
GOMF_RIBONUCLEOTIDE_BINDING	0.009	0.0218
GOBP_REGULATION_OF_HELICASE_ACTIVITY	0.024	0.022
GOMF_PEPTIDE_N_ACETYLTRANSFERASE_ACTIVITY	0.015	0.0221
GOMF_GAMMA_CATENIN_BINDING	0.032	0.0222
GOBP_POSITIVE_REGULATION_OF_MITOTIC_CELL_CYCLE	0.012	0.0222
GOMF_SOLUTE_CATION_ANTIPORTER_ACTIVITY	0.018	0.0223
GOCC_X_CHROMOSOME	0.018	0.0223
GOMF_ATP_DEPENDENT_ACTIVITY_ACTING_ON_RNA	0.015	0.0224
GOMF_TRANSFERASE_ACTIVITY_TRANSFERRING_PHOSPHORUS _CONTAINING_GROUPS	0.009	0.0224
GOMF_LYS63_SPECIFIC_DEUBIQUITINASE_ACTIVITY	0.021	0.0225
GOBP_NEGATIVE_REGULATION_OF_SMALL_MOLECULE _METABOLIC_PROCESS	0.012	0.0225
GOBP_PROTEIN_DEGLYCOSYLATION	0.025	0.0225
GOMF_CARBOHYDRATE_CATION_SYMPORTER_ACTIVITY	0.028	0.0225
GOBP_VESICLE_COATING	0.011	0.0226
GOMF_MAGNESIUM_ION_BINDING	0.009	0.0227
GOBP_REGULATION_OF_HISTONE_H3_K4_METHYLATION	0.012	0.0227
GOBP_POSITIVE_REGULATION_OF_UBIQUITIN_PROTEIN _TRANSFERASE_ACTIVITY	0.018	0.0228
GOBP_GLUTAMINE_METABOLIC_PROCESS	0.02	0.0229
GOBP_REGULATION_OF_CARDIAC_MUSCLE_CELL_ACTION_POTENTIAL	0.023	0.0229
GOBP_POSITIVE_REGULATION_OF_PROTEIN _LOCALIZATION_TO_CELL_PERIPHERY	0.014	0.0232
GOBP_ESTABLISHMENT_OF_CENTROSOME_LOCALIZATION	0.019	0.0233
GOBP_STRESS_GRANULE_ASSEMBLY	0.016	0.0234
GOBP_RESPONSE_TO_ARSENIC_CONTAINING_SUBSTANCE	0.012	0.0234
GOBP_REGULATION_OF_CIRCADIAN_RHYTHM	0.011	0.0235
GOMF_INTRAMEMBRANE_LIPID_TRANSPORTER_ACTIVITY	0.024	0.0235
GOMF_NUCLEAR_RECEPTOR_BINDING	0.014	0.0237

GOBP_REGULATION_OF_TELOMERE_CAPPING	0.013	0.024
GOMF_SECONDARY_ACTIVE_TRANSMEMBRANE _TRANSPORTER_ACTIVITY	0.014	0.024
GOBP_ESTABLISHMENT_OF_PROTEIN_LOCALIZATION_TO_VACUOLE	0.015	0.0241
GOBP_RIBOSE_PHOSPHATE_BIOSYNTHETIC_PROCESS	0.008	0.0241
GOBP_MODIFIED_AMINO_ACID_TRANSPORT	0.02	0.0242
GOBP_DESMOSOME_ORGANIZATION	0.048	0.0244
GOBP_NUCLEOSIDE_BISPHOSPHATE_METABOLIC_PROCESS	0.015	0.0244
GOBP_CIRCADIAN_RHYTHM	0.011	0.0246
GOBP_TOLL_LIKE_RECEPTOR_2_SIGNALING_PATHWAY	0.024	0.0246
GOCC_NUCLEAR_ENVELOPE	0.009	0.0246
GOBP_CELLULAR_RESPONSE_TO_GLUCOSE_STARVATION	0.01	0.0247
GOBP_TRICARBOXYLIC_ACID_METABOLIC_PROCESS	0.012	0.0248
GOBP_ACETYL_COA_BIOSYNTHETIC_PROCESS_FROM_PYRUVATE	0.025	0.0248
GOBP_NEGATIVE_REGULATION_OF_OXIDATIVE _STRESS_INDUCED_NEURON_DEATH	0.015	0.0249
GOBP_CELLULAR_RESPONSE_TO_FATTY_ACID	0.013	0.0249
GOBP_ACTIN_FILAMENT_NETWORK_FORMATION	0.024	0.025
GOBP_IMP_METABOLIC_PROCESS	0.027	0.0251
GOBP_REGULATION_OF_DNA_TEMPLATED_TRANSCRIPTION_IN _RESPONSE_TO_STRESS	0.018	0.0253
GOMF_NUCLEAR_EXPORT_SIGNAL_RECEPTOR_ACTIVITY	0.02	0.0253
GOBP_REGULATION_OF_GOLGI_ORGANIZATION	0.02	0.0254
GOCC_GOLGI_APPARATUS_SUBCOMPARTMENT	0.014	0.0254
GOBP_MICROTUBULE_ORGANIZING_CENTER_LOCALIZATION	0.015	0.0255
GOBP_REGULATION_OF_FAT_CELL_PROLIFERATION	0.021	0.0256
GOBP_L_GLUTAMATE_TRANSMEMBRANE_TRANSPORT	0.013	0.0258
GOBP_REGULATION_OF_CYTOPLASMIC_TRANSPORT	0.025	0.0259
GOBP_CARBOHYDRATE_TRANSPORT	0.013	0.026
GOBP_PROTEIN_TARGETING_TO_VACUOLE	0.019	0.0264
GOBP_FAT_CELL_PROLIFERATION	0.021	0.0264
GOCC_MITOTIC_SPINDLE_MIDZONE	0.026	0.0264
GOBP_BRANCHING_INVOLVED_IN_LABYRINTHINE _LAYER_MORPHOGENESIS	0.031	0.0265
GOBP_REGULATION_OF_MYELOID_CELL_APOPTOTIC_PROCESS	0.021	0.0265
GOBP_FORMATION_OF_TRANSLATION_PREINITIATION_COMPLEX	0.014	0.0267
GOBP_MANGANESE_ION_TRANSMEMBRANE_TRANSPORT	0.036	0.0269
GOMF_MANGANESE_ION_TRANSMEMBRANE_TRANSPORTER_ACTIVITY	0.036	0.0269
GOMF_PROTEIN_CONTAINING_COMPLEX_DESTABILIZING_ACTIVITY	0.012	0.0269
GOCC_GOLGI_CISTERNA	0.015	0.0269
GOBP_STRIATUM_DEVELOPMENT	0.025	0.0271
GOMF_SUGAR_TRANSMEMBRANE_TRANSPORTER_ACTIVITY	0.017	0.0272
GOBP_POSITIVE_REGULATION_OF_LAMELLIPODIUM_ORGANIZATION	0.021	0.0275
GOBP_RENAL_ABSORPTION	0.02	0.0275
GOBP_REGULATION_OF_CELLULAR_RESPONSE_TO_HYPOXIA	0.021	0.0277
GOBP_POSITIVE_REGULATION_OF_ANION_TRANSPORT	0.016	0.0277
GOBP_TRACHEA_MORPHOGENESIS	0.027	0.0277
GOMF_ACETYLGALACTOSAMINYLTRANSFERASE_ACTIVITY	0.023	0.0281
GOMF_P53_BINDING	0.011	0.0282
GOBP_POSITIVE_REGULATION_OF_TRANSFERASE_ACTIVITY	0.009	0.0283
GOBP_POSITIVE_REGULATION_OF_MRNA_SPLICING_VIA_SPLICEOSOME	0.014	0.0283
GOBP_MAINTENANCE_OF_BLOOD_BRAIN_BARRIER	0.028	0.0283
GOBP_PROTEIN_MONOUBIQUITINATION	0.013	0.0283
GOMF_NUCLEOBASE_CONTAINING_COMPOUND_TRANSMEMBRANE _TRANSPORTER_ACTIVITY	0.017	0.0284
GOBP_VESICLE_LOCALIZATION	0.01	0.0287
GOBP_NUCLEOTIDE_SUGAR_METABOLIC_PROCESS	0.022	0.0288

GOMF_ATPASE_COUPLED_INORGANIC_ANION_TRANSMEMBRANE_TRANSPORTER_ACTIVITY	0.032	0.0288
GOBP_POSITIVE_REGULATION_OF_PROTEIN_MODIFICATION_BY_SMALL_PROTEIN_CONJUGATION_OR_REMOVAL	0.009	0.0289
GOMF_LYS48_SPECIFIC_DEUBIQUITINASE_ACTIVITY	0.015	0.029
GOMF_UBIQUITIN_LIKE_PROTEIN_CONJUGATING_ENZYME_BINDING	0.021	0.0291
GOBP_POSITIVE_REGULATION_OF_CYTOKINESIS	0.022	0.0291
GOBP_SMALL_REGULATORY_NCRNA_PROCESSING	0.017	0.0291
GOBP_INTRACELLULAR_STEROL_TRANSPORT	0.019	0.0295
GOBP_O_GLYCAN_PROCESSING	0.035	0.0295
GOBP_REGULATION_OF_CELL_DIVISION	0.011	0.0298
GOBP_NUCLEOSIDE_MONOPHOSPHATE_METABOLIC_PROCESS	0.017	0.0298
GOMF_UBIQUITIN_CONJUGATING_ENZYME_BINDING	0.023	0.0299
GOMF_PROTEIN_TYROSINE_KINASE_ACTIVATOR_ACTIVITY	0.02	0.0299
GOBP_EPHRIN_RECEPTOR_SIGNALING_PATHWAY	0.016	0.03
GOMF_SECONDARY_ACTIVE_MONOCARBOXYLATE_TRANSMEMBRANE_TRANSPORTER_ACTIVITY	0.017	0.03
GOBP_RESPONSE_TO_MERCURY_ION	0.024	0.0301
GOMF_MOLECULAR_CONDENSATE_SCAFFOLD_ACTIVITY	0.02	0.0301
GOMF_RNA_POLYMERASE_II_CTD_HEPTAPEPTIDE_REPEAT_KINASE_ACTIVITY	0.024	0.0303
GOBP_REGULATION_OF_GLOMERULAR_FILTRATION	0.02	0.0303
GOCC_NUCLEAR_PERIPHERY	0.011	0.0304
GOMF_STRUCTURAL_CONSTITUENT_OF_NUCLEAR_PORE	0.026	0.0305
GOBP_UTP_METABOLIC_PROCESS	0.021	0.0307
GOBP_REGULATION_OF_RNA_EXPORT_FROM_NUCLEUS	0.018	0.0307
GOBP_MEMBRANE_FISSION	0.01	0.0311
GOBP_PROTEIN_EXIT_FROM_ENDOPLASMIC_RETICULUM	0.017	0.0312
GOBP_APICAL_JUNCTION_ASSEMBLY	0.014	0.0315
GOMF_PHOSPHOTRANSFERASE_ACTIVITY_PHOSPHATE_GROUP_AS_ACCEPTOR	0.011	0.0315
GOBP_PROTEIN_LOCALIZATION_TO_CYTOSKELETON	0.011	0.0315
GOBP_SPERMATID_NUCLEUS_DIFFERENTIATION	0.013	0.0316
GOBP_REGULATION_OF_VENTRICULAR_CARDIAC_MUSCLE_CELL_ACTION_POTENTIAL	0.03	0.0317
GOBP_INTEGRATED_STRESS_RESPONSE_SIGNALING	0.014	0.0318
GOCC_MITOCHONDRIA_ASSOCIATED_ENDOPLASMIC_RETICULUM_MEMBRANE	0.016	0.0318
GOBP_REGULATION_OF_RESPONSE_TO_ENDOPLASMIC_RETICULUM_STRESS	0.009	0.032
GOBP_ACETYL_COA_BIOSYNTHETIC_PROCESS	0.019	0.032
GOBP_GOLGI_TO_ENDOSOME_TRANSPORT	0.027	0.032
GOBP_PHENOL_CONTAINING_COMPOUND_BIOSYNTHETIC_PROCESS	0.011	0.0321
GOBP_GROWTH_HORMONE_RECEPTOR_SIGNALING_PATHWAY	0.021	0.0321
GOBP_INSULIN_METABOLIC_PROCESS	0.034	0.0321
GOCC_CLATHRIN_ADAPTOR_COMPLEX	0.012	0.0322
GOBP_ECTOPIC_GERM_CELL_PROGRAMMED_CELL_DEATH	0.018	0.0323
GOBP_BIOLOGICAL_PROCESS_INVOLVED_IN_SYMBIOTIC_INTERACTION	0.012	0.0324
GOBP_RETINAL_ROD_CELL_DIFFERENTIATION	0.022	0.0324
GOBP_PHOSPHATE_ION_TRANSMEMBRANE_TRANSPORT	0.014	0.0324
GOBP_PROTEIN_MANNOSYLATION	0.02	0.0325
GOBP_CELLULAR_RESPONSE_TO_IONIZING_RADIATION	0.011	0.0326
GOBP_PROTEIN_LOCALIZATION_TO_PHAGOPHORE_ASSEMBLY_SITE	0.013	0.0327
GOBP_PERK_MEDIATED_UNFOLDED_PROTEIN_RESPONSE	0.012	0.0328
GOBP_POLARIZED_EPITHELIAL_CELL_DIFFERENTIATION	0.014	0.0329
GOMF_NEUTRAL_AMINO_ACID_TRANSMEMBRANE_TRANSPORTER_ACTIVITY	0.02	0.0329
GOBP_REGULATION_OF_SISTER_CHROMATID_COHESION	0.021	0.0329

GOBP_REGULATION_OF_ORGANIC_ACID_TRANSPORT	0.011	0.0331
GOMF_RNA_POLYMERASE_II_COMPLEX_BINDING	0.013	0.0333
GOCC_GOLGI_CISTERNA_MEMBRANE	0.015	0.0334
GOMF_HEXOSAMINIDASE_ACTIVITY	0.016	0.0334
GOBP_REGULATION_OF_NUCLEAR_TRANSCRIBED_MRNA _POLY_A_TAIL_SHORTENING	0.019	0.0335
GOBP_INTERLEUKIN_1_MEDIATED_SIGNALING_PATHWAY	0.02	0.0336
GOBP_NEGATIVE_REGULATION_OF_LIPID_LOCALIZATION	0.014	0.0337
GOBP_CELL_DEATH_IN_RESPONSE_TO_OXIDATIVE_STRESS	0.012	0.0338
GOMF_UBIQUITIN_LIKE_PROTEIN_PEPTIDASE_ACTIVITY	0.014	0.0338
GOMF_EXOGENOUS_PROTEIN_BINDING	0.017	0.034
GOBP_REGULATION_OF_GOLGI_TO_PLASMA _MEMBRANE_PROTEIN_TRANSPORT	0.023	0.0341
GOBP_ORGANOPHOSPHATE_ESTER_TRANSPORT	0.013	0.0341
GOMF_MIRNA_BINDING	0.014	0.0341
GOMF_NUCLEOBASE_CONTAINING_COMPOUND_KINASE_ACTIVITY	0.014	0.0341
GOMF_PHOSPHATASE_ACTIVITY	0.008	0.0342
GOBP_POSITIVE_REGULATION_OF_ISOTYPE _SWITCHING_TO_IGG_ISOTYPES	0.03	0.0344
GOBP_MRNA_CLEAVAGE	0.018	0.0345
GOMF_NUCLEAR_RECEPTOR_COACTIVATOR_ACTIVITY	0.016	0.0345
GOBP_REGULATION_OF_NUCLEOCYTOPLASMIC_TRANSPORT	0.01	0.0346
GOBP_PURINE_CONTAINING_COMPOUND _TRANSMEMBRANE_TRANSPORT	0.015	0.0347
GOMF_ARYLSULFATASE_ACTIVITY	0.031	0.0348
GOBP_GOLGI_RIBBON_FORMATION	0.031	0.0348
GOBP_CELLULAR_RESPONSE_TO_ESTRADIOL_STIMULUS	0.01	0.0348
GOBP_RESPONSE_TO_IRON_ION	0.012	0.0349
GOBP_L_GLUTAMATE_IMPORT_ACROSS_PLASMA_MEMBRANE	0.023	0.0351
GOMF_BITTER_TASTE_RECEPTOR_ACTIVITY	0.026	0.0352
GOBP_SOMITOGENESIS	0.01	0.0352
GOBP_POSITIVE_REGULATION_OF_CELL_CELL_ADHESION _MEDIATED_BY_CADHERIN	0.027	0.0353
GOBP_TERPENOID_BIOSYNTHETIC_PROCESS	0.024	0.0354
GOBP_NEUTRAL_AMINO_ACID_TRANSPORT	0.019	0.0354
GOCC_TIGHT_JUNCTION	0.014	0.0356
GOBP_NUCLEAR_TRANSPORT	0.01	0.0357
GOBP_POSITIVE_REGULATION_OF_TRANSCRIPTION _ELONGATION_BY_RNA_POLYMERASE_II	0.01	0.0358
GOBP_RETINA_LAYER_FORMATION	0.015	0.0361
GOBP_COPII_COATED_VESICLE_BUDDING	0.013	0.0362
GOCC_APICAL_PLASMA_MEMBRANE	0.015	0.0362
GOBP_CENTROSOME_DUPLICATION	0.014	0.0362
GOBP_CRD_MEDIATED_MRNA_STABILIZATION	0.015	0.0362
GOBP_NEURAL_CREST_FORMATION	0.019	0.0363
GOBP_CELLULAR_HYPOTONIC_RESPONSE	0.031	0.0363
GOBP_REGULATION_OF_HYDROGEN_PEROXIDE_MEDIATED _PROGRAMMED_CELL_DEATH	0.012	0.0363
GOBP_ACTIVATION_OF_TRANSMEMBRANE_RECEPTOR _PROTEIN_TYROSINE_KINASE_ACTIVITY	0.024	0.0364
GOBP_MODULATION_BY_HOST_OF_SYMBIONT_PROCESS	0.013	0.0365
GOBP_PROTEIN_LOCALIZATION_TO_NUCLEAR_ENVELOPE	0.026	0.0365
GOBP_ENTRY_INTO_HOST	0.013	0.0366
GOBP_LYSOSOMAL_PROTEIN_CATABOLIC_PROCESS	0.017	0.0366
GOCC_MITOCHONDRIAL_TRICARBOXYLIC_ACID _CYCLE_ENZYME_COMPLEX	0.014	0.0366
GOCC_SPINDLE_MIDZONE	0.019	0.0366
GOMF_CATION_CHLORIDE_SYMPORTER_ACTIVITY	0.017	0.0367

GOBP_CORTICAL_ACTIN_CYTOSKELETON_ORGANIZATION	0.018	0.0367
GOMF_OXIDOREDUCTASE_ACTIVITY_ACTING_ON_THE _ALDEHYDE_OR_OXO_GROUP_OF_DONORS	0.019	0.0368
GOBP_RNA_TEMPLATED_DNA_BIOSYNTHETIC_PROCESS	0.011	0.0369
GOCC_EUKARYOTIC_TRANSLATION_INITIATION_FACTOR_4F_COMPLEX	0.012	0.0372
GOBP_ERBB_SIGNALING_PATHWAY	0.013	0.0373
GOBP_LUNG_MORPHOGENESIS	0.017	0.0375
GOBP_REGULATION_OF_GENE_EXPRESSION_BY_GENOMIC_IMPRINTING	0.019	0.0378
GOMF_NUCLEAR_ESTROGEN_RECEPTOR_BINDING	0.014	0.0379
GOBP_PROTEIN_LOCALIZATION_TO_CELL_CELL_JUNCTION	0.026	0.0383
GOBP_NEGATIVE_REGULATION_OF_LIPID_TRANSPORT	0.017	0.0386
GOBP_KERATINOCYTE_PROLIFERATION	0.015	0.0386
GOMF_TRANSMEMBRANE_RECEPTOR_PROTEIN _PHOSPHATASE_ACTIVITY	0.03	0.0387
GOBP_NEGATIVE_REGULATION_OF_GENE_EXPRESSION_EPIGENETIC	0.01	0.0387
GOBP_PROTEIN_DEMANNOSYLATION	0.024	0.0387
GOBP_PURINE_DEOXYRIBONUCLEOSIDE_MONOPHOSPHATE _METABOLIC_PROCESS	0.026	0.0388
GOMF_NUCLEAR_GLUCOCORTICOID_RECEPTOR_BINDING	0.026	0.0388
GOBP_PROTEIN_FOLDING_IN_ENDOPLASMIC_RETICULUM	0.017	0.0389
GOBP_REGULATION_OF_ANION_TRANSMEMBRANE_TRANSPORT	0.02	0.039
GOMF_CARBOHYDRATE_DERIVATIVE_TRANSMEMBRANE_TRANSPORTE	0.02	0.039
GOBP_PANCREAS_DEVELOPMENT	0.014	0.0391
GOBP_POSITIVE_REGULATION_OF_TRANSCRIPTION _BY_RNA_POLYMERASE_I	0.018	0.0391
GOMF_PROTEIN_BINDING_INVOLVED_IN_HETEROTYPIC _CELL_CELL_ADHESION	0.041	0.0392
GOBP_BIOLOGICAL_PROCESS_INVOLVED_IN_INTERACTION_WITH_HOST	0.011	0.0392
GOBP_REGULATION_OF_VITAMIN_METABOLIC_PROCESS	0.017	0.0395
GOBP_REGULATION_OF_STEROID_BIOSYNTHETIC_PROCESS	0.012	0.0397
GOBP_SUBPALLIUM_DEVELOPMENT	0.021	0.0399
GOCC_COPI_COATED_VESICLE_MEMBRANE	0.018	0.04
GOBP_INTRACELLULAR_TRANSPORT	0.007	0.0401
GOCC_BETA_CATENIN_DESTRUCTION_COMPLEX	0.014	0.0401
GOCC_COPI_COATED_VESICLE	0.017	0.0401
GOBP_REGULATION_OF_PLASMA_MEMBRANE_ORGANIZATION	0.025	0.0402
GOBP_ESTABLISHMENT_OF_ORGANELLE_LOCALIZATION	0.007	0.0408
GOBP_REGULATION_OF_NUCLEOBASE_CONTAINING _COMPOUND_TRANSPORT	0.014	0.0408
GOBP_GENE_SILENCING_BY_RNA	0.011	0.0409
GOMF_OXIDOREDUCTASE_ACTIVITY_ACTING_ON_THE_ALDEHYDE _OR_OXO_GROUP_OF_DONORS_NAD_OR_NADP_AS_ACCEPTOR	0.017	0.0409
GOCC_AMINOACYL_TRNA_SYNTHETASE_MULTIENZYME_COMPLEX	0.019	0.041
GOBP_POLYOL_TRANSMEMBRANE_TRANSPORT	0.024	0.0411
GOBP_NEGATIVE_REGULATION_OF_OXIDATIVE _STRESS_INDUCED_CELL_DEATH	0.012	0.0411
GOBP_PROTEIN_O_LINKED_GLYCOSYLATION_VIA_SERINE	0.025	0.0414
GOMF_PROTEIN_DEMETHYLASE_ACTIVITY	0.022	0.0414
GOMF_ABC_TYPE_XENOBIOTIC_TRANSPORTER_ACTIVITY	0.022	0.0415
GOBP_FRUCTOSE_6_PHOSPHATE_METABOLIC_PROCESS	0.027	0.0419
GOBP_REGULATION_OF_PROTEIN_DEUBIQUITINATION	0.014	0.042
GOBP_DICARBOXYLIC_ACID_METABOLIC_PROCESS	0.011	0.0423
GOCC_GOLGI_ASSOCIATED_VESICLE_MEMBRANE	0.013	0.0423
GOMF_DEUBIQUITINASE_ACTIVITY	0.014	0.0423
GOBP_PYRIMIDINE_RIBONUCLEOSIDE_TRIPHOSPHATE _METABOLIC_PROCESS	0.018	0.0424
GOBP_PLACENTA_BLOOD_VESSEL_DEVELOPMENT	0.016	0.0426
GOMF_HEXOSE_TRANSMEMBRANE_TRANSPORTER_ACTIVITY	0.018	0.0426

GOBP_DICARBOXYLIC_ACID_TRANSPORT	0.01	0.0427
GOMF_GLYCOSYLTRANSFERASE_ACTIVITY	0.011	0.0428
GOMF_ATP_DEPENDENT_ACTIVITY_ACTING_ON_DNA	0.018	0.0429
GOMF_RNA_POLYMERASE_II_CORE_PROMOTER _SEQUENCE_SPECIFIC_DNA_BINDING	0.012	0.0429
GOBP_NADP_METABOLIC_PROCESS	0.015	0.0432
GOBP_CYTOSKELETON_DEPENDENT_CYTOKINESIS	0.008	0.0432
GOBP_MITOCHONDRIAL_DNA_METABOLIC_PROCESS	0.013	0.0432
GOBP_GLIAL_CELL_APOPTOTIC_PROCESS	0.019	0.0433
GOMF_SULFURIC_ESTER_HYDROLASE_ACTIVITY	0.027	0.0435
GOMF_PEPTIDE_LYSINE_N_ACETYLTRANSFERASE_ACTIVITY	0.015	0.0435
GOMF_CORE_PROMOTER_SEQUENCE_SPECIFIC_DNA_BINDING	0.01	0.0435
GOBP_ECTODERM_DEVELOPMENT	0.018	0.0436
GOBP_POSITIVE_REGULATION_OF_CELL_DIVISION	0.012	0.0436
GOBP_CELLULAR_PIGMENTATION	0.009	0.0436
GOMF_ATP_DEPENDENT_PROTEIN_FOLDING_CHAPERONE	0.01	0.0437
GOCC_DNA_REPAIR_COMPLEX	0.017	0.0437
GOMF_RNA_7_METHYLGUANOSINE_CAP_BINDING	0.015	0.0437
GOBP_POSITIVE_REGULATION_OF_EPIDERMAL_GROWTH_FACTOR _ACTIVATED_RECEPTOR_ACTIVITY	0.033	0.0437
GOBP_UBIQUITIN_DEPENDENT_PROTEIN_CATABOLIC_PROCESS _VIA_THE_MULTIVESICULAR_BODY_SORTING_PATHWAY	0.009	0.0437
GOMF_IONOTROPIC_GLUTAMATE_RECEPTOR_BINDING	0.016	0.0438
GOBP_PROTEIN_TARGETING_TO_LYSOSOME	0.02	0.0439
GOBP_CHROMOSOME_LOCALIZATION_TO_NUCLEAR _ENVELOPE_INVOLVED_IN_HOMOLOGOUS _CHROMOSOME_SEGREGATION	0.022	0.0439
GOBP_REGULATION_OF_GLUCONEOGENESIS	0.014	0.0442
GOBP_VESICLE_BUDDING_FROM_MEMBRANE	0.012	0.0444
GOBP_CELL_CYCLE_G1_S_PHASE_TRANSITION	0.009	0.0444
GOBP_REGULATION_OF_EXOSOMAL_SECRETION	0.018	0.0444
GOMF_KINASE_ACTIVITY	0.009	0.0446
GOCC_APICAL_PART_OF_CELL	0.014	0.0446
GOBP_SISTER_CHROMATID_COHESION	0.019	0.0446
GOMF_PHOSPHOLIPID_TRANSPORTER_ACTIVITY	0.014	0.0447
GOMF_ACTIVE_ION_TRANSMEMBRANE_TRANSPORTER_ACTIVITY	0.011	0.0447
GOBP_LOCOMOTION_INVOLVED_IN_LOCOMOTORY_BEHAVIOR	0.024	0.0448
GOBP_REGULATION_OF_ANION_TRANSPORT	0.013	0.0451
GOCC_AP_1_ADAPTOR_COMPLEX	0.013	0.0451
GOBP_POSITIVE_REGULATION_OF_TRANSCRIPTION _BY_RNA_POLYMERASE_III	0.021	0.0451
GOBP_MRNA_CLEAVAGE_INVOLVED_IN_MRNA_PROCESSING	0.021	0.0454
GOBP_ANION_TRANSMEMBRANE_TRANSPORT	0.012	0.0455
GOMF_HEXOSYLTRANSFERASE_ACTIVITY	0.013	0.0457
GOMF_LIGASE_ACTIVITY_FORMING_CARBON_SULFUR_BONDS	0.012	0.046
GOCC_FLEMMING_BODY	0.013	0.046
GOBP_POSITIVE_REGULATION_OF_MRNA_METABOLIC_PROCESS	0.011	0.046
GOBP_REGULATION_OF_CHROMOSOME_CONDENSATION	0.025	0.0462
GOBP_TIGHT_JUNCTION_ORGANIZATION	0.013	0.0463
GOMF_PHOSPHATE_ION_BINDING	0.03	0.0464
GOBP_POSITIVE_REGULATION_OF_WOUND_HEALING	0.015	0.0468
GOBP_MORPHOGENESIS_OF_AN_EPITHELIAL_FOLD	0.021	0.0469
GOBP_REGULATION_OF_AMINO_ACID_TRANSMEMBRANE_TRANSPORT	0.02	0.047
GOMF_ORGANOPHOSPHATE_ESTER_TRANSMEMBRANE _TRANSPORTER_ACTIVITY	0.016	0.047
GOBP_ERAD_PATHWAY	0.01	0.0471
GOBP_PRO_B_CELL_DIFFERENTIATION	0.027	0.0476
GOBP_MIDBODY_ABSCISSION	0.015	0.0478

GOBP_ORGANIC_HYDROXY_COMPOUND_BIOSYNTHETIC_PROCESS	0.011	0.0478
GOBP_V_D_J_RECOMBINATION	0.022	0.0478
GOCC_TRANS_GOLGI_NETWORK	0.012	0.048
GOBP_BARBED_END_ACTIN_FILAMENT_CAPPING	0.025	0.048
GOBP_POSITIVE_REGULATION_OF_EXTRINSIC_APOPTOTIC _SIGNALING_PATHWAY_VIA_DEATH_DOMAIN_RECEPTORS	0.027	0.048
GOBP_PIGMENT_GRANULE_LOCALIZATION	0.013	0.0482
GOBP_INTRACELLULAR_PROTEIN_TRANSPORT	0.007	0.0483
GOBP_RESPONSE_TO_MUSCLE_ACTIVITY	0.01	0.0483
GOBP_CELLULAR_CARBOHYDRATE_METABOLIC_PROCESS	0.011	0.0483
GOMF_EPIDERMAL_GROWTH_FACTOR_RECEPTOR_BINDING	0.017	0.0484
GOBP_MYELOID_CELL_APOPTOTIC_PROCESS	0.02	0.0485
GOBP_CARBOHYDRATE_DERIVATIVE_METABOLIC_PROCESS	0.008	0.0486
GOBP_REGULATION_OF_CENTRIOLE_REPLICATION	0.016	0.0488
GOMF_PROTEIN_ARGININE_N_METHYLTRANSFERASE_ACTIVITY	0.015	0.049
GOBP_EPITHELIAL_CELL_CELL_ADHESION	0.02	0.0491
GOBP_POLYOL_CATABOLIC_PROCESS	0.012	0.0491
GOCC_APICAL_JUNCTION_COMPLEX	0.013	0.0492
GOBP_ASTRAL_MICROTUBULE_ORGANIZATION	0.02	0.0492
GOCC_CHROMATOID_BODY	0.024	0.0492
GOBP_ERYTHROCYTE_DEVELOPMENT	0.017	0.0493
GOBP_RESPONSE_TO_HEPATOCYTE_GROWTH_FACTOR	0.018	0.0494
GOCC_DENDRITE_TERMINUS	0.012	0.0495
GOMF_ORGANIC_ANION_TRANSMEMBRANE_TRANSPORTER_ACTIVITY	0.014	0.0496
GOBP_ORGANOPHOSPHATE_BIOSYNTHETIC_PROCESS	0.007	0.0496
GOMF_DYNEIN_COMPLEX_BINDING	0.014	0.0498
GOMF_PROTEIN_TYROSINE_PHOSPHATASE_ACTIVITY	0.009	0.0499
GOMF_TASTE_RECEPTOR_ACTIVITY	0.017	0.05
GOBP_AMP_BIOSYNTHETIC_PROCESS	0.027	0.05

Table.S3. List of differential KEGG pathways of NLNM

KEGG pathways in NLNM	logFC	P.Value
KEGG_ENDOMETRIAL_CANCER	0.023	0.0008
KEGG_NICOTINATE_AND_NICOTINAMIDE_METABOLISM	0.028	0.0021
KEGG_TYPE_II_DIABETES_MELLITUS	0.019	0.0088
KEGG_COLORECTAL_CANCER	0.016	0.0121
KEGG_ADHERENS_JUNCTION	0.023	0.0153
KEGG_RIBOFLAVIN_METABOLISM	0.027	0.0155
KEGG_UBIQUITIN_MEDIATED_PROTEOLYSIS	0.011	0.0155
KEGG_CYSTEINE_AND_METHIONINE_METABOLISM	0.016	0.0165
KEGG_ERBB_SIGNALING_PATHWAY	0.015	0.0197
KEGG_CITRATE_CYCLE_TCA_CYCLE	0.021	0.0252
KEGG_ALANINE_ASPARTATE_AND_GLUTAMATE_METABOLISM	0.02	0.0338
KEGG_GLYCEROLIPID_METABOLISM	0.012	0.0417
KEGG_SELENOAMINO_ACID_METABOLISM	0.015	0.0452
KEGG_CHRONIC_MYELOID_LEUKEMIA	0.013	0.0462
KEGG_NON_HOMOLOGOUS_END_JOINING	0.024	0.0476
KEGG_PANCREATIC_CANCER	0.014	0.0495

Footnote: GO items: Enriched KEGG pathways. LogFC: FC represents the folding change, that is, the ratio of the expression of LNM and NLNM. The logarithm is taken as the base of 2. Statistically significant when p-value is less than 0.05.

Table.S3. List of differential KEGG pathways of LNM

KEGG pathways in LNM	logFC	P.Value
KEGG_ARACHIDONIC_ACID_METABOLISM	0.022	0.017
KEGG_SYSTEMIC_LUPUS_ERYTHEMATOSUS	0.025	0.0389

Footnote: GO items: Enriched KEGG pathways. LogFC: FC represents the folding change, that is, the ratio of the expression of LNM and NLNM. The logarithm is taken as the base of 2. Statistically significant when p-value is less than 0.05.
